# Who Tends to Appreciate Atonal Music? Higher Perceived Personal Control Leads to an Increased Inclination to Prefer Atonal Music

**DOI:** 10.3390/ijerph19063248

**Published:** 2022-03-10

**Authors:** Junfeng Liu, Shen-Long Yang, Feng Yu

**Affiliations:** 1 Music Education Centre, School of Humanities and Social Science, Xi’an Jiaotong University, Xi’an 710049, China; liujunfeng@xjtu.edu.cn; 2 Institute of Social Psychology, School of Humanities and Social Science, Xi’an Jiaotong University, Xi’an 710049, China; 3 Department of Psychology, School of Philosophy, Wuhan University, Wuhan 430072, China

**Keywords:** atonal music, perceived control, need for structure, compensatory control theory

## Abstract

Research on the aesthetic experience of music has largely focused on tonal music, while relatively less is known about individuals’ differences in the aesthetic experience of atonal music. According to the compensatory control theory, we hypothesized that perceived personal control significantly and positively predicted individuals’ tendency to prefer atonal music, while the need for structure played a mediating role. The present research investigated who tends to prefer atonal music, and why. A sample of college students listened to atonal music and completed questionnaires on perceived personal control, the need for structure, and their aesthetic judgment of the music. Our analysis showed that individuals with higher perceived personal control exhibited a stronger tendency to prefer atonal music, compared with those who had lower perceived control; moreover, the need for structure played a mediating role between perceived control and aesthetic experience of atonal music. These results revealed which audience was suitable for atonal music and extended the explanatory scope of the compensatory control theory. The theoretical and practical implications and limitations of these findings are discussed.

## 1. Introduction

Norman Lebrecht, a famous contemporary British classical music critic, once commented on Schoenberg, stating that “One of the great achievements of the composer, Arnold Schoenberg, was his ability to empty every concert hall on the planet.” [1]. In the field of Western classical music in the 20th century, Arnold Schoenberg’s step toward atonal music shocked the entire epoch and inspired many avant-garde composers. In the late 19th century, an ever-increasing number of composers began to make compositional innovations, gradually dissolving the traditional Western tonal system. However, the most notable step was taken by Schoenberg, who abandoned the use of tonality and traditional keys. Furthermore, Schoenberg invented the twelve-tone technique, which treated the 12 pitch-classes as equivalent, thereby abolishing the existence of the central note [2]. This practice led to intense dissonance and a lack of tonal hierarchical structure in music compositions, which was known as “atonal music.” In addition to Schoenberg’s technique, subsequent composers tried to adopt more radical techniques, by using not only pitches, as Schoenberg did, but also transferring this approach to other musical parameters, such as dynamics, duration, rhythm, timbre, and form [3]. While these powerful musical innovations gradually became typical of Western art music, from the 20th and 21st centuries, they also became a huge aesthetic challenge for listeners [4].

Atonal music does not follow the rules of traditional harmony and, therefore, lacks both a clear tonal center and a pitch hierarchy. Rather than facilitating the listening process, this characteristic creates difficulties in some aesthetic aspects, such as a memory of melodies [5] and emotional responses [6]. As a result, atonal music has always been niche and does not attract as large an audience as earlier Western classical repertoires. Despite this, the high diversity and avant-garde represented by this musical style has earned it a loyal following [7]. This phenomenon suggests that there are individual differences in the aesthetic experiences of atonal music. In this regard, previous studies have drawn several conclusions. For instance, Wranning and Wetterin find that individuals with high levels of openness to different experiences showed greater appreciation for atonal harmony [8]. As an important personality trait, openness to different experiences was found to be a predictor of a listener experiencing a physiological reaction, also known as creating the feeling of chills, during such music listening [9]. In addition, a two-week quantitative and qualitative study finds that musically-trained participants showed higher emotional responses to atonal music than those without musical training [6].

However, empirical research on individual differences in the aesthetic experience of atonal music is still sparse, and to gain insight into the aesthetic psychology and practical applications of such music, more research is needed to anticipate who is more inclined to enjoy it. Atonal music is perceived to be structureless and disordered [10], and many previous studies show that perceived personal control affects individuals’ needs and preferences for structure and order [11]. Therefore, it seems a feasible perspective from which to explore why people like or dislike atonal music. The purpose of the current study was to investigate the predictive effect of perceived personal control on the inclination to prefer atonal music, a relationship that has not previously been explored.

### 1.1. The Perceived Lack of Structure and Disorderliness of Atonal Music

Tonal hierarchical organization is the typical paradigm of traditional Western tonal music [12]. In this hierarchical system, certain tones are more stable, prominent, and important than others. For example, the first tone of the scale, which is called the tonic, serves as a central note, repeatedly appears at structurally important musical positions [13], and represents the cognitive reference point [14]. All other tones of the scale are organized around the tonic, according to their different importance, thus representing a hierarchical order. This hierarchical structure has been shown to facilitate the processing speed of music [15], recognition and recall of melodies [16], and the formation of predictions [17]. However, atonal music dissolves this hierarchical structure by giving equal treatment to all tones within an octave. Although composers attempt to preserve structural coherence, for instance through operations on intervallic cells [18], the structure and order of atonal music are more difficult than those of tonal music for most listeners to perceive [6].

Owing to this characteristic of atonal music, previous studies find individual differences in people’s appreciation for it; the more people tend to break the rules and not rely on the established order, the more they may appreciate and like this kind of music. For example, the personality trait “openness to different experiences” has been found to be a predictor of appreciation for atonal harmony [8]. Moreover, research on curiosity has shown that exploratory behavior and “novelty seeking” are associated with biological rewards [19,20,21]. Generally, the ability to withstand uncertainty and eliminate the constraints of structure and order seems to be the key factor in forming an individual’s preference for atonal music. Previous studies find that perceived personal control is an important predictor of this need for structure [11,22].

### 1.2. Perceived Personal Control and the Need for Structure

Perceived personal control refers to the perception that individuals can predict, handle, and control events that occur in daily life [23]. Previous studies show that individuals with lower perceived personal control have a higher demand for the order and structure of the objective world, which can be manifested in many aspects. For example, those who have a lower sense of control showed an orientation preferring order-supplying products [24]. Researchers also found that people who felt a threat to their personal control preferred jobs that offered certainty [25] and were more likely to support political candidates who bring stability and order to society [26]. In addition, some ordered and structured theoretical ideas [27,28] and works of art [29] were found to be preferred by individuals with a lower sense of control. Those with higher levels of personal control, however, were less likely to feel the need for their objective environment to be ordered and structured.

These results show that the less personal control an individual has, the more likely their need for structure, which is defined as one’s tendency to prefer things that are structured, orderly, and predictable [30,31]. Comparatively, individuals with higher levels of personal control do not show this tendency. An influential explanation of this effect comes from the compensatory control theory. According to the theory, there is a basic human need to feel that the external world is clear, simple, certain, and orderly. Having control over the objective world can satisfy this need to a large extent, and serve as an important source for a sense of security [22,26]. However, in reality, people’s sense of control often faces threats, as many things are beyond our control. In this case, individuals will feel that the world has become disordered and uncertain, and therefore become anxious. As a psychological strategy to overcome this anxiety, they will exhibit an increased need to feel the order and certainty of the external world, as compensation for their lack of perceived control. Consequently, many studies find that people with a lower sense of control seek out objective order and structure in many ways [30,32], as mentioned above. Based on these conclusions, we speculate that, as a form of music that disrupts people’s orderly experience, atonal music precisely violates the need for structure of individuals with a lower sense of control, and therefore may be more ostracized by them. In contrast, individuals with higher levels of perceived control may be more receptive to the disruption of order, and thus, appreciate atonal music.

Therefore, it is reasonable to speculate that the appreciation of atonal music is also related to perceived personal control, and the need for structure may play a role as a psychological mechanism.

### 1.3. Atonal Music, Perceived Control, and the Need for Structure

Based on the existing evidence, we assume that there may be a mediating relationship between perceived control, the need for structure, and the aesthetic experience of atonal music. First, perceived control may predict the tendency to prefer atonal music. As opposed to the aesthetic experience of tonal music, the aesthetic purpose of atonal music is to overcome listeners’ auditory habits and stimulate auditory senses [33]. While tonal music brings a positive aesthetic experience, through the balance of structure, consonant harmony, and orderly hierarchy, atonal music may bring this experience through other paths, such as newness and novelty. However, the preferences for newness and novelty are not equally represented in everyone. For example, individuals with a higher need for structure may dislike atonal music, because its structure is more difficult to perceive; individuals with a lower need for structure, who are inclined to break the monotony and routine, are more likely to develop a preference for this kind of music. Previous studies also find that individuals with a lower need for structure have better cognitive flexibility and creativity [34,35].

Second, as mentioned above, many studies support the assertion that higher perceived personal control can predict a lower need for structure from the perspective of the compensatory control theory [12,22]. Therefore, it can be inferred that there is a relationship between perceived personal control, the need for structure, and the aesthetic experience of atonal music. When listening to atonal music, individuals with lower perceived personal control may be more inclined to seek clear structures; thus, they will probably encounter huge difficulties and challenges. However, the tendency to seek clear structures is not very strong in individuals with higher perceived control. This allows them to focus more on the novel auditory stimuli of atonal music, thus resulting in a better aesthetic experience. Therefore, we hypothesized that individuals with higher perceived control will have a better aesthetic experience of atonal music (Hypothesis 1, H1) with the need for structure playing a mediating role (Hypothesis 2, H2).

### 1.4. The Current Study

To understand the relationship between perceived control, need for structure, and the aesthetic experience of atonal music, questionnaires were used to measure perceived control as an independent variable and aesthetic experience of atonal music as a dependent variable. We also measured the need for a structure to verify its mediating role. Using a cross-sectional study design, we recruited Chinese college students to complete the questionnaire.

## 2. Method

### 2.1. Participants

The current study was conducted among 434 college students at a university in Xi’an, China. Six participants were excluded for missing data, yielding a final sample of 428 participants (252 men and 176 women), aged 17 to 23 years (*M* = 18.57, *SD* = 0.84).

### 2.2. Materials and Measures

#### 2.2.1. Music Materials

The chosen musical piece was the first of Schoenberg’s Three Piano Pieces, Op. 11, which is representative of his work. This musical piece broke away from the constraints of the traditional tonal hierarchy, representing Schoenberg’s formal step towards atonal music. Owing to the abandonment of traditional tonal systems and the extensive use of dissonant tones, the structure of this musical work has been shown to be more difficult to identify than that of a tonal piece [6].

#### 2.2.2. Perceived Personal Control

We used the revised Chinese version of the Sense of Control Scale [36], originally developed by Lachman and Weaver [23], to measure perceived personal control. The scale includes 12 items such as, “I can do almost anything I set my mind to” and “What happens in my life is often out of my control” (reverse scoring). Participants responded on a Likert scale ranging from 1 (strongly disagree) to 7 (strongly agree). The Cronbach’s alpha for the scale was 0.76.

#### 2.2.3. Need for Structure

This was assessed using the revised Chinese version of the Need for Structure Scale [37], originally developed by Neuberg and Newsom [31]. The scale consists of 11 items such as “It makes me upset to enter a situation without knowing what I can expect from it” and “I’m not bothered by things that disrupt my daily routine” (reverse scoring). Answer options ranged from 1 (strongly disagree) to 6 (strongly agree). The Cronbach’s alpha for the scale was 0.77.

#### 2.2.4. Aesthetic Experience of Atonal Music

In accordance with previous studies, we measured the participants’ aesthetic experience based on their self-reported preference and liking of the music [38,39,40]. After measuring perceived personal control and need for structure, participants wore headphones to listen to the music and were asked how much they enjoyed what they had just heard (liking), and how interested they were in it (interest). Both questions used a 1 (low) to 6 (high) Likert-type scale.

### 2.3. Procedure

The participants completed the study in a classroom. All participants were fully informed that their anonymity was guaranteed, why the research was being conducted, and how their data would be used. Informed consent was obtained from the participants. Then, their perceived personal control and need for structure were measured. Next, they were asked to wear headphones and listen to the music described above, which they could play on their phones by scanning a QR code. After listening, they completed a questionnaire that measured their aesthetic experiences. Finally, the participants answered questions about their gender and age. To thank them for their time, each participant who provided a valid response received ¥5.

### 2.4. Statistical Analysis

The SPSS 21.0 program was used to perform statistical analyses on the collected data.

## 3. Results

### 3.1. Common Method Bias Test

Harman’s single-factor test was used to examine common method variance [41]. The results showed that the first factor accounted for 17.06% of the total variance, and did not explain most of the variance (<40%). Thus, there was no obvious methodological bias in this study.

### 3.2. Descriptive Statistics and Correlations

As shown in Table 1, perceived personal control was positively correlated with the aesthetic experience of atonal music, specifically with liking, *r*(426) = 0.11, *p* = 0.018, and interest, *r*(426) = 0.13, *p* = 0.006. However, perceived control was negatively correlated with the need for structure, *r*(426) = −0.13, *p* = 0.01, which was, in turn, negatively correlated to the aesthetic experience of atonal music, specifically with liking, *r*(426) = −0.15, *p* = 0.002, and interest, *r*(426) = −0.12, *p* = 0.016, thus providing the basis for mediation analysis among these variables.

### 3.3. Mediation Effect of the Need for Structure

**Figure 1:**
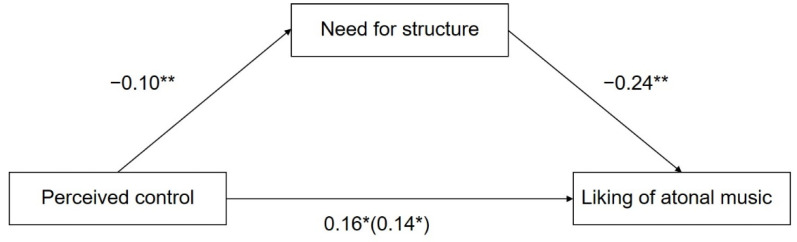
Mediation model of the need for structure in the relationship between perceived control and liking of atonal music. Path values are the path coefficients with standard errors. * *p* < 0.05. ** *p* < 0.01.

We used the PROCESS macro for IBM SPSS 19.0 (Model 4), developed by Hayes, to evaluate the mediating effect of the need for structure between perceived personal control and the aesthetic experience of atonal music [42]. First, we considered liking atonal music as the dependent variable. The results showed that the total effect of perceived control on liking atonal music was significant (β = 0.16, t = 2.37, *p* = 0.018), 95% Confidence Interval (CI) (0.03, 0.30). As shown in Figure 1 and Table 2, perceived control negatively predicted the need for structure (β = −0.10, t = −2.59, *p* = 0.01), 95% CI (−0.18, −0.02), and the need for structure, in turn, negatively predicted liking of atonal music (β = −0.24, t = −2.80, *p* = 0.005), 95% CI (−0.40, −0.07). The residual direct effect was still significant (β = 0.14, t = 2.02, *p* = 0.04), 95% CI (0.004, 0.27). Therefore, the need for structure played a mediating role in the link between perceived control and liking atonal music (indirect effect = 0.02), 95% CI (0.01, 0.06), and the proportion of the mediating effect was 14.68%.

**Figure 2:**
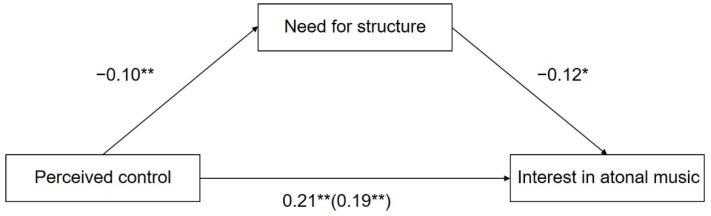
Mediation model of the need for structure in the relationship between perceived control and interest in atonal music. Path values are the path coefficients with standard errors. * *p* < 0.05. ** *p* < 0.01.

Next, we used atonal music as the dependent variable. The total effect of perceived control on interest in atonal music was significant (β = 0.21, t = 2.76, *p* = 0.006), 95% CI (0.06, 0.36). As shown in Figure 2 and Table 3, perceived control negatively predicted the need for structure (β = −0.10, t = −2.59, *p* = 0.01), 95% CI (−0.18, −0.02), and the need for structure, in turn, negatively predicted interest in atonal music (β = −0.12, t = −2.10, *p* = 0.036), 95% CI (−0.39, −0.01). The residual direct effect was still significant (β = 0.19, t = 2.49, *p* = 0.01), 95% CI (0.04, 0.34). Therefore, the need for structure played a mediating role in the link between perceived control and interest in atonal music (indirect effect = 0.02), 95% CI (0.003, 0.06), and the proportion of the mediating effect was 9.52%.

## 4. Discussion

This study examined the relationship between perceived personal control and the aesthetic experience of atonal music, as well as its underlying mechanism. The results showed that individuals with higher perceived personal control exhibited a stronger tendency to prefer atonal music, compared with those who had lower perceived control; moreover, the need for structure was shown to play a mediating role between perceived control and aesthetic experience of atonal music, which supported Hypotheses 1 and 2. People with higher perceived control are less motivated to seek order and structure while listening and can focus more on the novelty of atonal music. Conversely, people with lower perceived control rely more on things with order and structure; thus, it is more difficult to experience pleasure when listening to atonal music. The results of this study are consistent with our prediction that the mediating mechanism of the need for structure, combined with the compensatory control theory, provides an explanation for this phenomenon.

It is worth noting that the mean scores for liking and interest in atonal music are relatively low. One previous study finds that listeners have lower emotional responses to atonal music than to tonal music and that this effect is strongest in individuals with no musical training [6]. Moreover, familiarity has always been considered as a predictive variable of music preference [43]. Considering the abovementioned aesthetic barriers of atonal music, and that the current study’s participants have no musical training and are completely unfamiliar with atonal music, the floor effect apparent in the data seems reasonable and does not undermine the main findings.

Why do so many people dislike atonal music? Atonal music is an auditory adventure, and the pleasure obtained from listening to it may be more pronounced in certain individuals than in others. Previous studies have explored the predictive effect of personality traits on the aesthetic experiences of atonal music. Openness to different experiences is regarded as an important indicator of appreciation for this kind of music [33]. Individuals with higher levels of openness to different experiences prefer reflective and complex musical genres [9], enjoy musical forms outside the mainstream of popular and rock music [44], and exhibit a greater appreciation for atonal harmony [8]. Compared with the aforementioned studies, the novelty of this study is shown in the following two aspects. First, we investigated the prediction effect of perceived personal control on the aesthetic experience of atonal music, which has not previously been explored. Second, by introducing the mediating variable of the need for structure, we found that individuals who were more likely to break inherent rules and relied less on structure and order showed a stronger tendency to appreciate atonal music, which verified previous studies on openness to different experiences to some extent [9,45]. A previous study also found that people who lack a sense of control prefer fluent stimulation [46]. This effect can also be explained by mechanisms of compensatory control. This conclusion and the findings of the present study support each other.

In general, this study adds theoretical value to the literature in the following aspects: First, we found that perceived control was a predictor of a tendency to prefer atonal music. The appreciation of atonal music varies among individuals. However, previous studies have not adequately investigated this issue. This study found a new explanation for the individual differences. Second, from the perspective of perceived control, this study also has implications for future research. Previous studies focused more on the effects of perceived control on personal mental and physical health [47]. However, this study found that perceived control can also predict individual art appreciation preferences. From this perspective, future studies can explore the influence of the sense of control on individual art appreciation. Third, the present study found that the need for structure played a mediating role in the relationship between perceived control and the aesthetic experience of atonal music, which not only clarified why personal control could positively predict one’s tendency to prefer atonal music but also provided new evidence on the compensatory control theory. Consequently, we can deduce that, for individuals who lack a sense of control, their compensatory control manifests itself in many ways. In addition to religious beliefs [26], consumption [48], and political attitudes [32], which have been studied by researchers, compensatory control is also reflected in artistic appreciation.

The findings of this study have several practical implications. Through the investigation of individual differences in the aesthetic experience of atonal music, a portrait of the audience that is suitable for this kind of music can be drawn. Previous studies find that individuals from higher social classes may experience a higher level of perceived personal control, owing to their affluent living environment, personal freedom, and higher social status [49]. Hence, advertisements for concerts of atonal music could be targeted at this group. In addition, research shows that if those who lack a sense of control can perceive order in some way, their tendency to demand order and structure will decrease [26]. Therefore, making the typesetting of the program and the layout of the concert scene appear more orderly and structured may be an effective intervention to improve the aesthetic experience of atonal music.

There are, however, some limitations in the current study, which can be explored in future research. First, our findings were derived from correlation analysis, therefore, experimental manipulations need to be applied in future research to obtain causal results. Second, although the mediating role of the need for structure was verified by this study, the mediating effect size was not large, indicating that there are other psychological mechanisms between perceived control and aesthetic experience of atonal music, which should be addressed in future research. Finally, the participants of this study were mainly non musically trained adolescents; thus, a larger sample from the non-student population or the musically trained population may be needed to obtain more robust conclusions in future research.

## 5. Conclusions

This research explored the relationship between perceived personal control and aesthetic experience of atonal music, as well as the underlying psychological mechanisms, by conducting cross-sectional surveys. The results showed that individuals’ perceived control positively predicted their inclination to enjoy atonal music. Our findings indicate that there are individual differences in the appreciation of atonal music, and individuals who lack a sense of control find it more difficult to obtain positive aesthetic experiences from atonal music owing to their need for structure.

## Figures and Tables

**Table 1:** Descriptive analysis and correlations.

	*M*	*SD*	1	2	3	4
1. Perceived personal control	4.56	0.76	1			
2. Need for structure	4.09	0.62	–0.13 **	1		
3. Liking of atonal music	2.82	1.09	0.11 *	–0.15 **	1	
4. Interest in atonal music	2.91	1.22	0.13 **	–0.12 *	0.70 ***	1

Note. * *p* < 0.05. ** *p* < 0.01. *** *p* < 0.001.

**Table 2:** Mediating effect test of the need for structure.

	Effect Value	Boot *SE*	Lower Boot CI	Upper Boot CI
Total effect of perceived control on the liking of atonal music	0.16	0.07	0.03	0.30
Direct effect of perceived control on the liking of atonal music	0.14	0.07	0.004	0.27
Indirect effect of perceived control on the liking of atonal music	0.02	0.01	0.01	0.06

Note. Boot standard error, lower Boot confidence interval (CI), and upper Boot CI refers to the standard error, lower limit, and upper limit of the 95% CI, respectively, of effects estimated by the percentile Bootstrap method with deviation correction.

**Table 3:** Mediating effect test of need for structure.

	Effect Value	Boot *SE*	Lower Boot CI	Upper Boot CI
Total effect of perceived control on interest in atonal music	0.21	0.08	0.06	0.36
Direct effect of perceived control on interest in atonal music	0.19	0.08	0.04	0.34
Indirect effect of perceived control on interest in atonal music	0.02	0.01	0.003	0.06

Note. Boot standard error, lower Boot confidence interval (CI), and upper Boot CI refers to the standard error, lower limit, and upper limit of the 95% CI, respectively, of effects estimated by the percentile Bootstrap method with deviation correction.

## Data Availability

Data will be provided by the authors on request.
